# A Distributed Whole Genome Sequencing Benchmark Study

**DOI:** 10.3389/fgene.2020.612515

**Published:** 2020-12-01

**Authors:** Richard D. Corbett, Robert Eveleigh, Joe Whitney, Namrata Barai, Mathieu Bourgey, Eric Chuah, Joanne Johnson, Richard A. Moore, Neda Moradin, Karen L. Mungall, Sergio Pereira, Miriam S. Reuter, Bhooma Thiruvahindrapuram, Richard F. Wintle, Jiannis Ragoussis, Lisa J. Strug, Jo-Anne Herbrick, Naveed Aziz, Steven J. M. Jones, Mark Lathrop, Stephen W. Scherer, Alfredo Staffa, Andrew J. Mungall

**Affiliations:** ^1^Canada’s Michael Smith Genome Sciences Centre, BC Cancer Research Institute, Provincial Health Services Authority, Vancouver, BC, Canada; ^2^McGill Genome Centre, McGill University, Montreal, QC, Canada; ^3^The Centre for Applied Genomics, The Hospital for Sick Children and University of Toronto, Toronto, ON, Canada; ^4^Canada’s Genomics Enterprise (CGEn), The Hospital for Sick Children, Toronto, ON, Canada

**Keywords:** whole genome sequencing, genome, benchmark, informatics, comparison, variant

## Abstract

Population sequencing often requires collaboration across a distributed network of sequencing centers for the timely processing of thousands of samples. In such massive efforts, it is important that participating scientists can be confident that the accuracy of the sequence data produced is not affected by which center generates the data. A study was conducted across three established sequencing centers, located in Montreal, Toronto, and Vancouver, constituting Canada’s Genomics Enterprise (www.cgen.ca). Whole genome sequencing was performed at each center, on three genomic DNA replicates from three well-characterized cell lines. Secondary analysis pipelines employed by each site were applied to sequence data from each of the sites, resulting in three datasets for each of four variables (cell line, replicate, sequencing center, and analysis pipeline), for a total of 81 datasets. These datasets were each assessed according to multiple quality metrics including concordance with benchmark variant truth sets to assess consistent quality across all three conditions for each variable. Three-way concordance analysis of variants across conditions for each variable was performed. Our results showed that the variant concordance between datasets differing only by sequencing center was similar to the concordance for datasets differing only by replicate, using the same analysis pipeline. We also showed that the statistically significant differences between datasets result from the analysis pipeline used, which can be unified and updated as new approaches become available. We conclude that genome sequencing projects can rely on the quality and reproducibility of aggregate data generated across a network of distributed sites.

## Introduction

The global sequencing market is valued at approximately $10 billion^1^. To date, more than 500,000 human genomes have been sequenced^2^ and deposited in public databases as part of previous large-scale genome projects (Auton et al., 2015; Turro et al., 2020), personal genome projects (Beck et al., 2018; Reuter et al., 2018; Jeon et al., 2020) or sizeable aggregation projects across larger populations (Karczewski et al., 2019). The genomes of another two million individuals are expected to be sequenced under current projects^3^ ^,4^. To date, such data have been used to increase understanding of the underlying genetic architecture in disease (Yuen et al., 2017; Bailey et al., 2018; Priestley et al., 2019; Pleasance et al., 2020; Trost et al., 2020) and are increasingly being used in clinical genetics settings (Stavropoulos et al., 2016; Lionel et al., 2018).

For large-scale projects where expansive data are to be collected across populations, the resources of many institutions may be pooled to meet sequencing capacity demands, as well as to satisfy possible jurisdictional requirements, ethno-cultural and anthropological considerations (Knoppers et al., 2014), as well as ethical or legal restrictions on sample transfer (Mascalzoni et al., 2015), or requirements for grant funds to be spent locally. As genome sequences become increasingly used as the foundational biological reference point for national precision medicine initiatives, multi-site participation will only increase (Stark et al., 2019). In such projects, it is important to identify and quantify any differences in results that may arise due to different methodological and analytical procedures used across sites. While there are methods to evaluate and correct for batch effects once data have been generated (Tom et al., 2017; Baskurt et al., 2020) for whole genome sequencing projects, genetic variants for example cannot be reproducibly called if the appropriate reads are not sampled on a given sequencing instrument. Therefore, generation of consistently comparable data is preferred.

To facilitate the evaluation of whole genome assays, the genome in a bottle (GIAB) consortium combines sequence data from multiple centers along with results from several variant calling algorithms to provide consensus variant calls and importantly, regions of confident genotyping for each of the model samples (Zook et al., 2016). The consortium enables sequencing centers to routinely assess the precision and sensitivity of single nucleotide variants (SNVs) and insertion and deletions (Indels) detected in their analyses by sequencing GIAB reference samples (Cleary et al., 2015).

In order to prepare to support national genome sequencing initiatives of the highest quality for sharing in open-science databases (Rahimzadeh et al., 2016) three GIAB reference cell lines were sequenced in triplicate at each of Canada’s Michael Smith Genome Sciences Centre at BC Cancer in Vancouver, The Centre for Applied Genomics at The Hospital for Sick Children in Toronto, and the McGill Genome Centre in Montreal. Importantly, all processes were performed using current best practice approaches as determined at each center, to allow us to accurately assess differences observed under production conditions. Assessing the results for the 81 openly accessible whole genome data sets generated from combinations of samples, replicates, sequencing center, and analysis center allowed us to rank the variables in order of the associated variability in results. Our results inform our own, and any other multi-site projects, on how to collectively yield the most accurate genome sequence data and genetic variant calls.

## Methods

**FIGURE 1:**
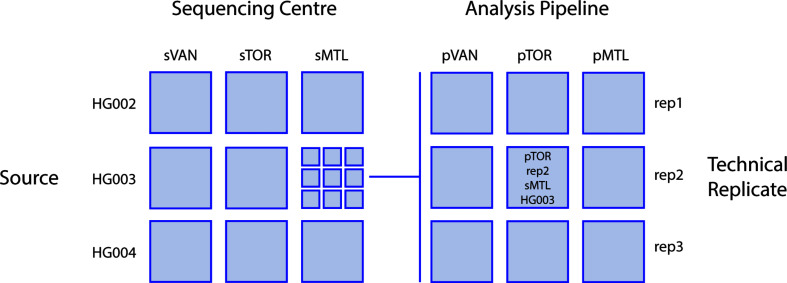
Study design. Sequence data for three different reference DNA sample sources (the Ashkenazim trio; son HG002, father HG003, and mother HG004) were generated in triplicate at each center then analyzed by each center for a total of 81 total combinations of sequencing center, DNA Source, analysis pipeline, and technical replicate. Sequencing centers (s) and analysis pipelines (p): VAN, Vancouver; MTL, Montreal; TOR, Toronto.

Each of the three sequencing centers used the Illumina HiSeq X technology to generate short-read genome sequence data of at least 30X coverage, using DNA from three GIAB reference cell lines (see below). These resulting 27 datasets were then processed through the bioinformatic pipelines in use at each center to create 81 datasets defined by four variables: unique cell line, replicate, sequencing center, and analysis pipeline. Figure 1 provides an overview of the combinatorial study design, which leveraged the benchmark data provided by the GIAB consortium (Zook et al., 2016). The genome sequence data are submitted to the NCBI SRA database under accession SRP278908.

### Samples

The samples used were from the National Institute of Standards and Technology (NIST) reference material 8392. These are further described as “Human DNA for Whole-Genome Variant Assessment (Family Trio of Eastern Europe Ashkenazi Jewish Ancestry) (HG002, HG003, HG004)” (Zook et al., 2016). DNAs were obtained from large homogenized growths of B lymphoblastoid cell lines from the Human Genetic Cell Repository at Coriell Institute for Medical Research. To eliminate any potential variability from differences in DNA preparation between sites, the samples sequenced at each site were aliquots from the same primary preparation.

### PCR-Free Whole Genome Sequencing

Each center performed DNA quality control (QC), library construction, and sequencing steps following their own standard procedures (summarized in Table 1), some of which are the same, with other components being different. Of note, two different PCR-free library preparation kits and DNA input amounts were employed, and target insert sizes and input starting amounts of DNA also differed across centers. As indicated, all sequencing was performed on Illumina HiSeq X instruments. While 1% PhiX spike-in was used in both Montreal and Toronto, Vancouver used its standard method of including a plasmid-based sample tracking spike-in.

**TABLE 1:** Laboratory methods as performed at each of the three centers.

		**Sequencing center (s)**		
		**sVAN**	**sMTL**	**sTOR**
gDNA QC assays	Integrity	Agarose gel	Agarose gel or Tapestation	Agarose gel or Tapestation
	Quantification	Qubit or Quant-it DNA HS assays	Qubit DNA HS assay	Qubit DNA HS assay
	Purity	Not applicable	A260/280 between 1.8 and 2.0	A260/280 between 1.8 and 2.0
WGS library construction	PCR-free library prep kit	NEB paired-end sample prep Premix kit	Illumina TruSeq PCR-free library prep kit	Illumina TruSeq PCR-free library prep kit
	Input DNA amount (ng)	500	500	700
	DNA fragmentation	Covaris LE220	Covaris LE220	Covaris LE220
	Target size range (bp)	300–400	300	400
	Size selection	PCRClean DX (Aline Biosciences)	Ampure beads (Beckman Coulter)	Ampure beads (Beckman Coulter)
Library QC	Library validation (sizing)	Agilent Bioanalyzer DNA high sensitivity assay	Agilent Bioanalyzer DNA high sensitivity assay	Agilent Bioanalyzer DNA high sensitivity assay
	Library validation (quantification)	KAPA qPCR library quant kit	KAPA qPCR library quant kit	KAPA qPCR library quant kit
Sequencing	Sequencer (reads)	HiSeq X (2 × 150)	HiSeq X (2 × 150)	HiSeq X (2 × 150)
	Genomes (library) per lane	1	1	1
	Spike-in controls	Tracking plasmid (Moore et al., 2020)	1% PhiX	1% PhiX

*sVAN, Vancouver; sMTL, Montreal; sTOR, Toronto.*

### Genetic Variant Calling and Informatics

Analysis methods for germline, PCR-free genomes that were performed at each center are reported in Table 2. All analyses were performed against each center’s chosen human genome reference assembly based on NCBI’s Genome Reference Consortium human build 37 (GRCh37), each performing alignments using BWA mem (Li, 2013). Of note, two centers (Montreal and Toronto) employed GATK 3.7+ and associated best-practice workflows (McKenna et al., 2010; DePristo et al., 2011; Van der Auwera et al., 2013) while Vancouver used Strelka 2 (Kim et al., 2018) without any explicit steps for base recalibration or Indel realignment.

**TABLE 2:** Informatics tools and settings employed at each center.

	**Sequencing center analysis pipeline (p)**		
	**pVAN**	**pMTL**	**pTOR**
Reference	Hg19a^6^	hs37d5^7^	hs37d5^7^
Read trimming	Custom trimmed to 150 bp	Skewer 0.2.2	Not applicable
Alignment	BWA mem 0.7.6a-M	BWA mem 0.7.12	BWA mem 0.7.12
BAM sorting	Sambamba 0.5.5	Sambamba 0.6.6	Picard 2.5.0 SortSam
BAM duplicate marking	Sambamba 0.5.5	Sambamba 0.6.6	Picard 2.5.0 MarkDuplicates
BAM calibration	Not applicable	GATK 3.8 BQSR and IR	GATK 3.7.0 BQSR and IR
Variant calling	Strelka 2.9.2	GATK 3.8 HaplotypeCaller	GATK 3.7.0 HaplotypeCaller
Variant filtering	Not applicable	Not applicable	GATK 3.7.0 VQSR

*pVAN, Vancouver; pMTL, Montreal; pTOR, Toronto. ^6^https://www.bcgsc.ca/downloads/genomes/9606/hg19/1000genomes/bwa_ind/genome/README.GRCh37-lite^7^http://www.imsbio.co.jp/RGM/R_rdfile?f=BSgenome.Hsapiens.1000genomes.hs37d5/man/package.Rd&d=R_BC*

To assess differences observed in the data in advance of variant calling, aligned reads from each pipeline were processed with Picard^5^, Qualimap (García-Alcalde et al., 2012), and SAMtools (Li et al., 2009) to identify quality differences. Variant calling results were assessed using version 3.6.2 of RTGTools vcfeval (Cleary et al., 2015) using release 3.3.2 of the GIAB references for HG002, HG003, and HG004.

## Results

Assessing each of the 81 BAM files for quality, we detected some notable differences in the data yielded by each center. The average read coverage across the 81 datasets was 36.5X, with both the lowest (30.9X) and highest (42.1X) coverage for a single lane of data coming from Montreal’s sequencing pipeline. Mean insert sizes were consistently lower in the data from Montreal, whose data had both more AT dropout and less GC dropout than data from the other centers (Supplementary Figure 1). While non-uniformity of read depth likely has little impact on the identification of SNVs, it can have notable effects on the sensitivity and specificity of CNV detection from whole genome sequence data, particularly when using read-depth based methods (Trost et al., 2019).

### Concordance Against Benchmark Data

The corresponding variant calls for each of the 81 BAM sequence files were compared to available benchmark data, and across data sets. Although there were significant differences in the raw data metrics, the primary focus for this project was the final concordance of resulting variant calls. When comparing results to the available benchmark data, final VCF files from all combinations of unique cell line, replicate, sequencing center, and analysis pipeline yielded sensitivity measures above 98.9% and precision values above 99.5%. Supplementary Figure 2 shows the full set of 81 sensitivity, precision, and F1 (model accuracy) values. Overall, the analysis pipelines from Montreal and Toronto, both of which employ the GATK based pipeline, had consistently higher sensitivity and lower precision than Vancouver, which employs the Strelka2 based pipeline. There was also a higher variance in F1 scores at Montreal where the results for the second replicate of HG003 and HG004 yielded reduced sensitivity in comparison to the other sets while the precision remained high. The sequencing results that generated consistently lower sensitivity had mean genome coverage numbers of 31.6X and 30.9X, while all others from the same center had mean coverage of 37.3X or higher. The raw data for the low coverage samples also had the highest estimated base error rates of the samples from the same lab (Supplementary Table 1).

### Intersection of Genetic Variant Calls

In addition to the need for production of high-quality genetic variant calls across a network of centers, equally important is that the variants called within each center must also be as consistent and reproducible as possible. SNVs and Indels generated for each of the three samples were intersected to assess the level of difference between pipeline configurations (Supplementary Table 2). To achieve this, we treated each of the samples independently, and for each we held two of the three remaining variables (sequencing center, analysis center, and replicate) constant to evaluate the amount of change observed when considering just one variable. For example, for sample HG002 sequenced in Montreal and analyzed at Toronto, three sets of results were produced (one for each of the three replicates). Those three sets of results were intersected to evaluate the level of discordance across sequencing replicates. This type of analysis was repeated 27 times to cover all the combinations of sample, sequencing center, and analysis center to generate a distribution of expected differences due exclusively to the replicates.

**FIGURE 2:**
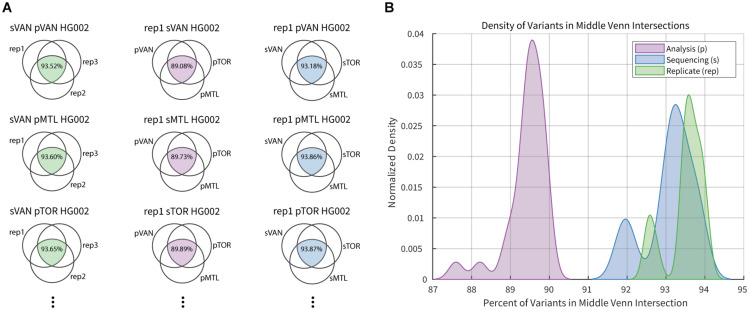
Example of intersection analysis for select HG002 datasets. **(A)** Column 1 (green) Venn diagrams compare three replicate sequence datasets generated in Vancouver (sVAN) and analyzed by all three center’s pipelines. Column 2 (purple) Venn diagrams compare the results from the three analysis pipelines for replicate 1 (rep1) of HG002 sequenced in Vancouver (sVAN), Montreal (sMTL) or Toronto (sTOR). Column 3 (blue) Venn diagrams compare results from the three sequencing centers for replicate 1 of HG002 analyzed by the three pipelines in Vancouver (pVAN), Montreal (pMTL) and Toronto (pTOR). In total there were 81 3-way intersections completed. **(B)** Percentage of variants common across sequencing replicates (green), analysis pipelines (purple) and sequencing centers (blue) are summarized in the density plots.

A summary of the intersection analysis is presented in Figure 2, where the fraction of variants from each three-way comparison that are common to all three sets was collected. The fraction of variant calls that were common across sequencing replicates (median = 93.6) was slightly higher than that for the sequencing center (median = 93.2), and significantly greater (Mann–Whitney-Wilcoxon test, *p*-value = 1.027e-15) than that for the analysis pipeline (median = 89.5). While investigating each variable, there were multiple datasets with higher discordance than the common distribution for the 27 data points contributing to each curve. In each case, the more variable results occurred when comparing variant calls that included the second technical replicates from Montreal, which had an average coverage near 30X while other datasets from the same center had closer to 39X coverage. Historically genome sequencing studies have used a threshold of 30X coverage although this has, in part, been driven by sequencing costs and target density when loading flowcells. As expected, our results confirm the benefit of deeper sequence coverage for accurate variant calling. As sequencing costs continue to decrease, in principle, we expect to generate higher average genome coverage and therefore, higher variant calling accuracy.

## Discussion

Our results indicate that performing whole genome sequencing, using the technology platform tested across multiple sites is an acceptable approach when trying to maximize sample size, for example, for large-scale population or disease studies. We presented a framework for testing multiple variables controlled by the sequencing centers, and found the most significant differences when different analysis pipelines were implemented. This underlines the robustness of the library preparation protocols, sequencing and imaging applied in this study, which minimizes experimental errors identified in short read sequencing (Robasky et al., 2014). We have made our data publicly available for additional testing.

Much of the evaluation was completed by comparing variant calls to benchmark data provided by the GIAB Consortium, where millions of true positive SNVs and Indels are known for each sample and, critically, large regions of confident non-variant positions allowing for the assessment of precision. However, it should be noted that these regions do not cover all classes of genetic variants, nor the entire genome, and studies such as this one cannot assess the precision or quality of variant calls within the missing regions. For example, version 3.3.2 of the available benchmark data for HG002 lists confident genotype information for 2.358 Gb of the genome but does not contain any information for variants on the X or Y chromosomes. Moreover, copy number and structural genetic variation datasets were not yet examined (Scherer et al., 2007). It is also important to consider the source of the DNA sample (Trost et al., 2019), which can influence the quality and amount of input DNA used for sequencing. Each of these factors may in turn impact all aspects of data generated, in particular when long-read technologies are used rather than the short-read sequencing presented here (Wang et al., 2019; Thibodeau et al., 2020).

There were two samples in the second replicate run, originating from one site, that yielded lower average coverage (31.6X and 30.9X) than its other samples, all of which had an average coverage greater than 37X. As expected, these particular samples had the lowest variant calling sensitivity suggesting that an average genome coverage nearing 30X may compromise sensitivity in germline studies, and higher coverage could be recommended. The average coverage numbers, however, do not explain all of the differences that are observed. In Figure 2, the low coverage samples cause the peaks at the lower end of each distribution while the larger distributions show that the choice of analysis pipeline can have a large impact on the consistency of variant results, as has been described by us and others (Craig et al., 2016; Chen et al., 2019; Kumaran et al., 2019). A consistent analysis pipeline is expected to improve across-center consistency by up to 5%, assuming that the variance among replicates represents the maximal reproducibility across datasets.

Since each of the three participating centers developed their pipelines largely independently (although there were some ongoing, cross-site projects sharing concepts), it was encouraging to find that overall genome variant calling results were both of high quality and consistent between sites. Our study did reveal minor differences in approaches, such as the selection of the version of the reference sequence used; two centers used an identical reference (hs37d5), but the third typically used hg19a (see section “Methods”).

In summary, the employment of different standard analysis pipelines was thus determined as the main source of variation between datasets generated by the three centers. Fortunately, this aspect of the sequencing process can be easily controlled, either prospectively, or retrospectively. Major technology developments and operational guidelines for this purpose have been put forth in recent years, motivated precisely by reproducibility challenges in genomic data generation. Here, we add to this growing body of literature, arriving at recommendations for our own path forward, in which we suggest the three centers implement containerization using Singularity (Kurtzer et al., 2017) and portable workflows using workflow definition language (WDL) (Voss et al., 2017) in each local high-performance computing facility. With these capabilities in place, our distributed sequencing network is poised to generate consistent, high-quality, whole-genome datasets for national, as well as international-scale, projects.

## Data Availability Statement

The datasets presented in this study can be found in online repositories. The names of the repository/repositories and accession number(s) can be found below: https://www.ncbi.nlm.nih.gov/, SRP278908.

## Author Contributions

AM, RC, SS, RW, and JW wrote the manuscript. AS and AM supervised the study. RC, RE, JW, NB, MB, EC, JJ, RM, KM, NM, SP, MR, BT, RW, JR, LS, JA-H, NA, AS, and AM are members of CGEn’s technical experts committee that conceived of and executed the study. SJ, ML, NA, and SS are the scientific directors of CGEn and oversaw all aspects of this project. All authors reviewed and approved the final manuscript.

## Conflict of Interest

SS is on the Scientific Advisory Boards of Deep Genomics and Population Bio and intellectual property arising from his research held at The Hospital for Sick Children is licensed by Athena Diagnostics and Lineagen. The Center for Applied Genomics, directed by SS and The Hospital for Sick Children has benefited from joint relationships with Illumina and other suppliers, but none of these arrangements have impacted the studies described in this paper. SS holds the Canadian Institutes of Health Research (CIHR) GlaxoSmithKline Endowed Chair for Genome Sciences at The Hospital for Sick Children and University of Toronto. The remaining authors declare that the research was conducted in the absence of any commercial or financial relationships that could be construed as a potential conflict of interest.

## Footnotes

1 https://www.grandviewresearch.com/industry-analysis/next-generation-sequencing-market

2 https://www.forbes.com/sites/matthewherper/2017/01/09/illumina-promises-to-sequence-human-genome-for-100-but-not-quite-yet/#f5df45c386d2

3 https://ec.europa.eu/digital-single-market/en/news/germany-joins-1million-genomes-initiative

4 https://www.nih.gov/news-events/news-releases/nih-funds-new-all-us-research-program-genome-center-test-advanced-sequencing-tools

5 http://broadinstitute.github.io/picard

## Supplementary Material

Supplementary Figure 1 Quality assessment of the 81 BAM files. For all four plots Ashkenazim trio DNA samples are listed on x-axis. Mean X coverage achieved for the genomes **(A)**, adenine and thymine percentage, or AT, dropout rate **(B)**, average insert size in base pairs **(C)**, and guanine and cytosine, or GC, percentage dropout rate **(D)**.

Supplementary Figure 2 Sensitivity, precision, and F1 values for the 81 datasets. Fractional values are provided for each comparison; F1 measure (gray bars), sensitivity (pink squares), and specificity (blue squares). Sequencing centers (s) and analysis pipelines (p): VAN, Vancouver; MTL, Montreal; TOR, Toronto. Reference DNA sample sources (the Ashkenazim trio; son HG002, father HG003, and mother HG004).
