# Supplementary figures and images for: A Distributed Whole Genome Sequencing Benchmark Study
Source: Front Genet. 2020 Dec 1;11:612515. doi: 10.3389/fgene.2020.612515 (PMC7736078; doi:10.3389/fgene.2020.612515)

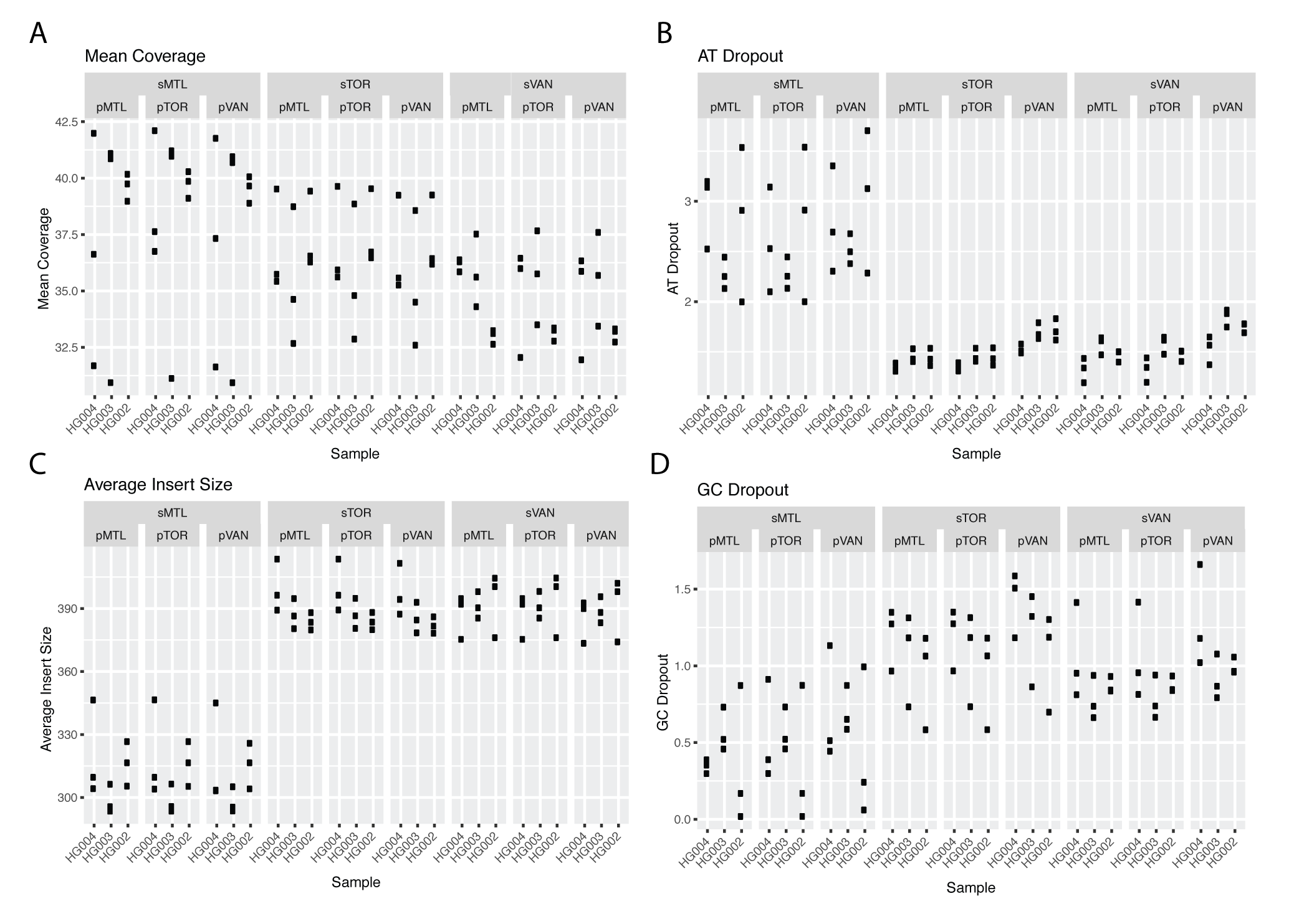

Supplement: Supplementary Figure 1 — Quality assessment of the 81 BAM files. For all four plots Ashkenazim trio DNA samples are listed on x-axis. Mean X coverage achieved for the genomes (A), adenine and thymine percentage, or AT, dropout rate (B), average insert size in base pairs (C), and guanine and cytosine, or GC, percentage dropout rate (D). [file Image_1.TIF]

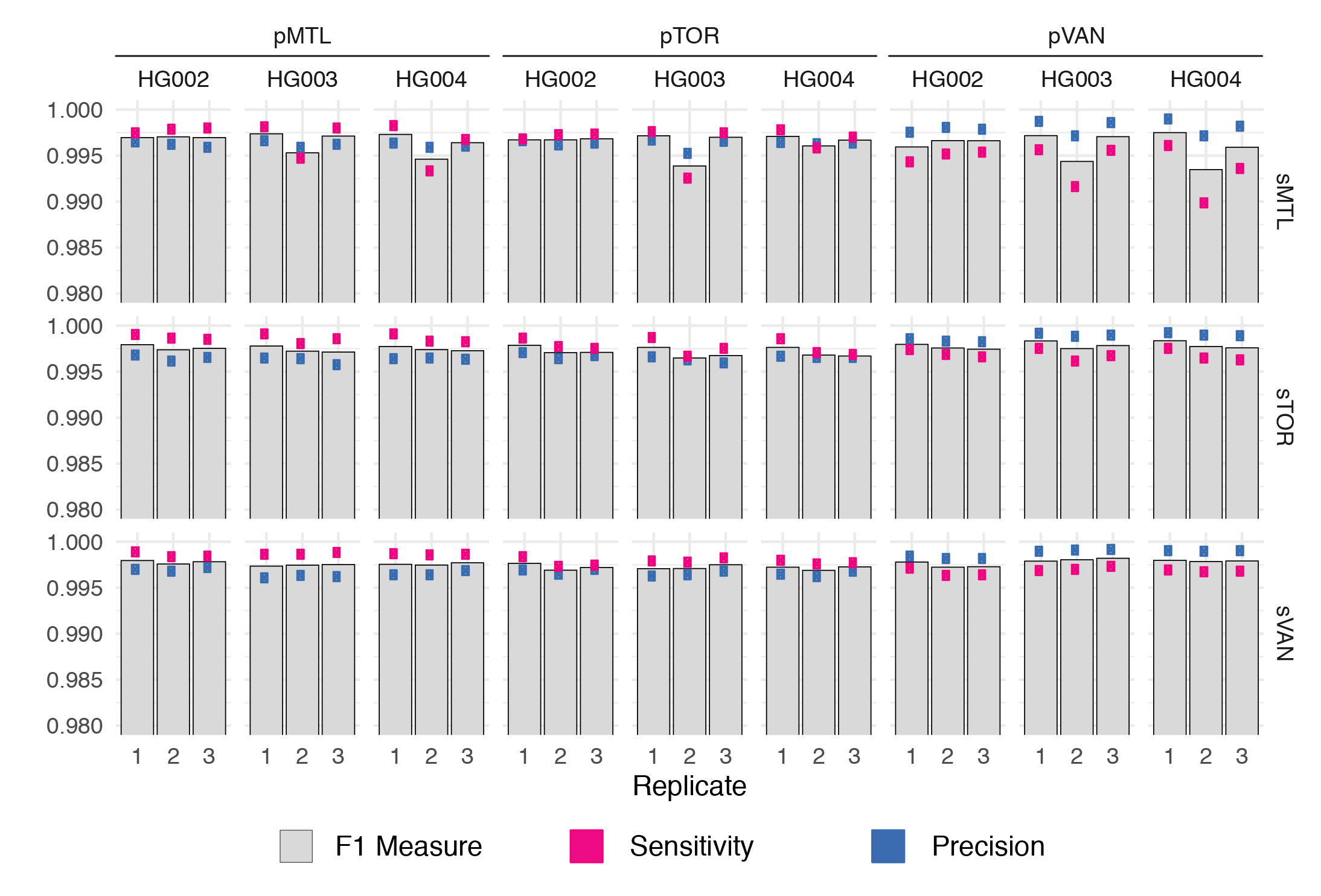

Supplement: Supplementary Figure 2 — Sensitivity, precision, and F1 values for the 81 datasets. Fractional values are provided for each comparison; F1 measure (gray bars), sensitivity (pink squares), and specificity (blue squares). Sequencing centers (s) and analysis pipelines (p): VAN, Vancouver; MTL, Montreal; TOR, Toronto. Reference DNA sample sources (the Ashkenazim trio; son HG002, father HG003, and mother HG004). [file Image_2.jpg]
